# Regional Haemodynamic and Metabolic Coupling in Infants

**DOI:** 10.3389/fnhum.2021.780076

**Published:** 2022-02-04

**Authors:** Maheen F. Siddiqui, Paola Pinti, Sarah Lloyd-Fox, Emily J. H. Jones, Sabrina Brigadoi, Liam Collins-Jones, Ilias Tachtsidis, Mark H. Johnson, Clare E. Elwell

**Affiliations:** ^1^Centre for Brain and Cognitive Development, Birkbeck College, University of London, London, United Kingdom; ^2^Department of Psychology, University of Cambridge, Cambridge, United Kingdom; ^3^Department of Development and Social Psychology, University of Padua, Padua, Italy; ^4^Department of Information Engineering, University of Padua, Padua, Italy; ^5^Department of Medical Physics and Biomedical Engineering, University College London, London, United Kingdom

**Keywords:** metabolism, fNIRS (functional near infrared spectroscopy), neurovascular coupling, brain specialization, neurodevelopment, mitochondria, social brain, brain metabolic imaging

## Abstract

Metabolic pathways underlying brain function remain largely unexplored during neurodevelopment, predominantly due to the lack of feasible techniques for use with awake infants. Broadband near-infrared spectroscopy (bNIRS) provides the opportunity to explore the relationship between cerebral energy metabolism and blood oxygenation/haemodynamics through the measurement of changes in the oxidation state of mitochondrial respiratory chain enzyme cytochrome-c-oxidase (ΔoxCCO) alongside haemodynamic changes. We used a bNIRS system to measure ΔoxCCO and haemodynamics during functional activation in a group of 42 typically developing infants aged between 4 and 7 months. bNIRS measurements were made over the right hemisphere over temporal, parietal and central cortical regions, in response to social and non-social visual and auditory stimuli. Both ΔoxCCO and Δ[HbO_2_] displayed larger activation for the social condition in comparison to the non-social condition. Integration of haemodynamic and metabolic signals revealed networks of stimulus-selective cortical regions that were not apparent from analysis of the individual bNIRS signals. These results provide the first spatially resolved measures of cerebral metabolic activity alongside haemodynamics during functional activation in infants. Measuring synchronised changes in metabolism and haemodynamics have the potential for uncovering the development of cortical specialisation in early infancy.

## Introduction

Specialisation of function by region is one of the key organising principles of the human brain. Indirect evidence suggests that in some cases specialisation emerges gradually over the course of development (Johnson, 2011). Such observations are critical for testing theoretical predictions about the degree to which core principles of brain organisation are present at birth or emerge through experience, and how that process takes place. However, direct evidence from neuroimaging studies remains sparse due to the technical and practical challenges involved in imaging the developing human brain (de Roever et al., 2018). In adults, functional magnetic resonance imaging (fMRI) is the most widely employed technique to obtain non-invasive, spatially resolved measures of brain activity. This technique infers localisation of specific cognitive measures by detecting regional changes in the ratio of oxygenated/deoxygenated haemoglobin (also referred to as blood oxygenation levels). More specifically, when neural activity in particular brain regions increases, there is a concomitant increase in neural oxygen demand (Kim and Filosa, 2012). Groups of nearby vascular, neural and glial cells detect this demand and as a result produce metabolites that act on blood vessels to produce regionally-specific increases in blood flow and consequently the changes in blood oxygenation levels that are measured by fMRI, in a process referred to as neurovascular coupling (NVC) (Abbott et al., 2006). The oxygen that is supplied to neural and glial cells is used to convert adenosine diphosphate (ADP) to adenosine triphosphate (ATP) in the mitochondria to produce cellular energy. However, fMRI poses significant practical limitations for use in awake infants, as it requires them to be immobile and supine. Further, measurements of changes in blood oxygenation do not allow us to disentangle the developmental changes in specialisation of brain function that may arise from developmental changes in neurovascular coupling (Harris et al., 2011). Previous studies have demonstrated that there are indeed developmental changes in a number of components of NVC; namely vasculature (Chen et al., 2014; Hall et al., 2014; Hill et al., 2015; Iadecola, 2017), vascular reactivity (Chen et al., 2014), cerebral metabolic rate of oxygen consumption (CMRO_2_) (Chugani et al., 1987) and regional cerebral blood flow (rCBF) (Chiron et al., 1992; Takahashi et al., 1999), which may all affect NVC. Thus, techniques are required that allow measurement of different components of the NVC pathway such as metabolism and blood oxygenation in awake infants.

Functional near-infrared spectroscopy (fNIRS) is an alternative technique that provides measures of changes in concentration of oxygenated (Δ[HbO_2_]) and deoxygenated haemoglobin (Δ[HHb] with up to 1 cm spatial resolution (Quaresima and Ferrari, 2019). Over the last twenty years, fNIRS has been employed increasingly to investigate neurodevelopment in a diverse range of research areas such as language (Telkemeyer et al., 2011; Issard and Gervain, 2017; Minagawa et al., 2019) and infant social cognition (Wilcox et al., 2013; Urakawa et al., 2015; McDonald and Perdue, 2018; Pirazzoli et al., 2019; Bulgarelli et al., 2020). fNIRS can be used to measure brain activity when infants are awake and upright, allowing investigation of neuronal activation in naturalistic contexts. fNIRS has already provided confirmatory evidence that haemodynamic responses might be different in infancy. Animal studies using infant rats of an equivalent age to human newborns reported inverted responses to somatosensory stimulation (i.e., an increase in ΔHHb and a decrease in ΔHbO_2_) which gradually transitioned into a classical response in adulthood (Kozberg et al., 2013). Human studies have also shown heterogeneity in the haemodynamic response to functional activation in infants using both fNIRS (Watanabe et al., 2012; Issard and Gervain, 2018) and fMRI (Martin et al., 1999; Born et al., 2000, 2002; Yamada et al., 2000; Arichi et al., 2012). Other human infant studies observe a similar but delayed infant haemodynamic response (HRF) relative to the adult response (Homae et al., 2006; Grossmann et al., 2008; Lloyd-Fox et al., 2009; Hyde et al., 2010; Ichikawa et al., 2013; Wilcox et al., 2013), with the delay gradually reducing across development (Lloyd-Fox et al., 2017). Such evidence reinforces the importance of dissociating developmental changes in brain activation from developmental changes in the associated haemodynamic response (Issard and Gervain, 2018).

In the present study, we report the use of a novel technique, referred to as broadband near-infrared spectroscopy (bNIRS), that allows non-invasive measurement of cellular energy metabolism alongside haemodynamics/oxygenation in awake infants. The dynamics of these measures remains largely unexplored during neurodevelopment due to the lack of availability of suitable techniques, particularly for use with infants. bNIRS therefore allows us the opportunity to gain insight into NVC mechanisms in the developing human brain. This technique uses a broader range of optical wavelengths than fNIRS, providing the spectral resolution to measure the oxidation state of mitochondrial respiratory chain enzyme cytochrome-c-oxidase – a direct measure of cellular energy metabolism (Bale et al., 2016). CCO is located in the inner mitochondrial membrane and is the terminal electron acceptor in the electron transport chain (ETC). It is responsible for more than 95% of cellular oxygen metabolism. Briefly, CCO has a copper A redox centre that has a unique absorption spectra in its oxidised and reduced forms in the near-infrared region (NIR) (Bale et al., 2016). The difference between the reduced and oxidised forms can be used as a measure of the change in the oxidation status of CCO (referred to here as ΔoxCCO). bNIRS measures of ΔoxCCO exhibit higher brain specificity than HbO_2_ and HHb (de Roever et al., 2016) and additionally, this measure correlates with Phosphorus Magnetic Resonance Spectroscopy markers of mitochondrial function (Bainbridge et al., 2014). In adult functional activation studies, multichannel bNIRS has been used to measure spatial localisation of ΔoxCCO (Phan et al., 2016a). This measure may therefore provide an intermediate measure linking metabolism to oxygenation/haemodynamic changes to neuronal activity, thereby enabling insight into NVC in early infancy.

In a previous study, we demonstrated the feasibility of bNIRS in infants during functional activation using a single channel bNIRS system (Siddiqui et al., 2017). This was the first reported study to obtain non-invasive measures of changes in cerebral cellular energy metabolism alongside haemodynamics in the developing human brain using bNIRS. Single channel measurements, however, do not allow spatial localisation of brain function and multiple channels across the head are required to obtain spatially resolved responses. We now present the use of a multichannel bNIRS system to measure spatially resolved changes in ΔoxCCO, alongside haemodynamic changes, over the visual cortex and the right temporal cortex in 4-to-7-month-old typically developing infants. Social/non-social video stimuli were used to measure cortical activation. These stimuli have been used extensively in previous infant studies (Lloyd-Fox et al., 2009, 2014; Jones et al., 2015; Siddiqui et al., 2017) and produce a robust haemodynamic response over the temporal cortex, providing an appropriate context to explore the relationship between haemodynamics and metabolism in infancy. We expected activation in the visual cortex related to visual processing of stimuli. We hypothesised that the task-averaged changes in ΔoxCCO would show differential responses to the two stimuli which would be expected to be coupled with haemodynamic changes.

Additionally, we investigated the relationship between haemodynamics and metabolism using a method that has previously been used to explore this relationship in adults using fMRI-PET data (Shokri-Kojori et al., 2019) and broadband NIRS data (Pinti et al., 2021). This method works by computing two indices which reflect the coupling between haemodynamics and metabolism. These parameters are: (1) the relative power (rPWR) which indicates the extent to which haemodynamic and metabolic activity are matched and (2) the relative cost (rCST) which indicates the extent of the mismatch between metabolic and haemodynamic activity, i.e., the extent to which metabolic activity exceeds or lags behind haemodynamic activity. High rPWR values are expected in brain areas that are metabolically demanding and indicate higher metabolic and brain (haemodynamic) activity relative to other brain regions. High rCST values have been observed in brain regions associated with high-level cognitive functions. This has been attributed to the employment of less efficient metabolic pathways such as aerobic glycolysis (or non-oxidative glucose metabolism). It has also been suggested that high rCST indicates higher glia-to-neuron ratio (Vaishnavi et al., 2010; Shokri-Kojori et al., 2019). We expect to see proportional haemodynamic and metabolic changes in the healthy infant brain.

## Materials and Methods

### Participants

Forty-two 4-to-7-month-old infants participated in the study (22 males and 20 females, mean age: 179 ± 16 days). All parents volunteered to participate in the study and provided written, informed consent. The study protocol was approved by the Birkbeck Ethics Committee. The infants were from varying ethnic backgrounds, predominantly White (British/non-British). 80% of the infants in the study were White while 20% of the infant were from ethnic backgrounds. All the infants who participated were screened prior to recruitment into the study and parents were requested to provide information regarding gestational age at birth, pregnancy/birth complications, detailed medical history of the mother and infant including if the infant needed any special care after delivery, and any familial history of neurodevelopmental disorders. Infants were included in the study if they were born at term between 37 and 40 weeks’ gestation, had no known developmental disorders and there was no familial history of neurodevelopmental disorders.

### Experimental Procedure

**FIGURE 1:**
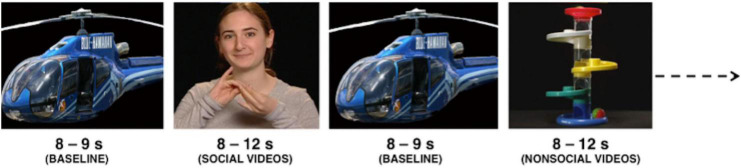
Order of stimulus presentation.

A 35-in screen was used to display the experimental stimuli while the infants were seated on their parent’s lap at a viewing distance of approximately 65 cm. All experimental stimuli were designed using Psychtoolbox in Matlab (Mathworks, United States). The social condition consisted of a variety of full-colour video clips of female actors performing/singing nursery rhymes such as “incy wincy spider” and “wheels on the bus”. The non-social condition consisted of dynamic videos of moving mechanical toys. Both conditions consisted of an audio component which matched the visual. These videos have been used previously in infant EEG studies (Jones et al., 2015) and are similar to stimuli used in NIRS studies (Lloyd-Fox et al., 2009). Both social and non-social experimental conditions were presented for a varying duration between 8 and 12 s. The baseline condition (see also Lloyd-Fox et al., 2009) consisted of static images of different types of transport, for example helicopters and cars, which were presented randomly for a pseudorandom duration of 1–3 s each, for a total of 8 s. Following this, a fixation cross in the shape of a ball or flower appeared in order to draw the infant’s attention back to the screen in case the infant had become bored during the baseline period. The following experimental condition was then presented only once the infant started looking at the fixation cross. Figure 1 shows the order of stimulus presentation. The study began with a rest period (10 s minimum) in order to draw the infant’s attention toward the screen, during which the infant was shown shapes in the four corners of the screen. Following this, the baseline and experimental conditions were alternated until the infant became bored or fussy. Alerting sounds were occasionally played during the baseline period to draw the infant’s attention back to the screen.

### Data Acquisition and Array Placement

**FIGURE 2:**
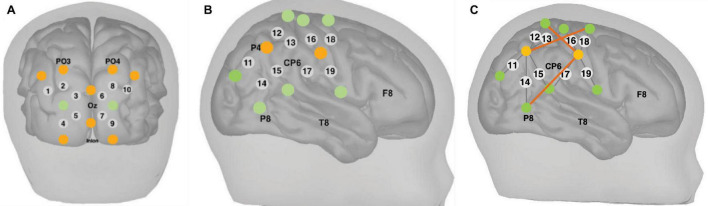
Locations of NIRS channels (grey circles) and locations of the sources (orange circles) and detectors (green circles) fibres relative to 10/20 locations indicated on the figure for channels over the **(A)** occipital cortex and **(B)** right hemisphere. The grey lines show each source-detector pairing that forms each channel. The source-detector separation was 2.5 cm. **(C)** Locations of long-distance channels indicated as dark orange lines between the sources and detectors for the purpose of image reconstruction, in channels over the right hemisphere. The source-detector separation for the long-distance channels was 4.3 cm.

**FIGURE 3:**
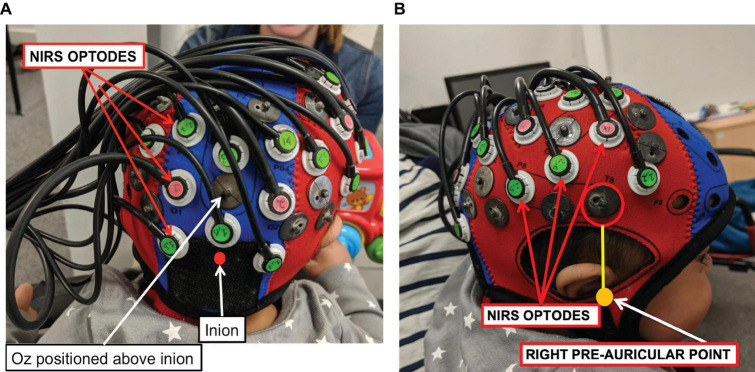
A NIRS-MRI age-appropriate co-registration map (Lloyd-Fox et al., 2014) was used to align the lower front of the array (marked with a red circle) with the right pre-auricular anatomical landmark. The bNIRS optodes are indicated, the green optodes are detectors while the pink ones are sources. Panel **(A)** shows the posterior view and panel **(B)** shows the lateral view.

Measurements of ΔoxCCO, Δ[HbO_2_] and Δ[HHb] were made using a broadband system which was developed at University College London (Phan et al., 2016b). Briefly, the bNIRS system consisted of two light sources, each fitted with a 50W halogen light bulb (Phillips) with an axial filament needed to emit the broadband NIR enhanced spectrum. Light from the sources was directed to the subject by use of customised bifurcated optical fibres (Loptek, Germany) which allowed each light source to split into two pairs of light sources. This formed a total of four light sources at the subject-end and each pair of light sources were controlled by a time multiplexing mechanism whereby one pair of light sources was on every 1.4 s. The system also consisted of fourteen fibre detectors at the subject-end which were connected to two spectrometers, seven for each spectrometer (in-house developed lens spectrographs and PIXIS512f CCD cameras (Princeton Instruments). The fibre detector configuration along with the light sources, formed a total of nineteen measurement channels. Nine of these channels were positioned over the right hemisphere and had a source-detector separation of 2.5 cm, as shown in Figure 2, relative to 10/20 locations. The remaining ten channels were positioned over the occipital cortex. The channels were placed on the head using custom-built, 3-D printed arrays and headgear, over the occipital cortex and the right hemisphere. The headgear is shown positioned on an infant in Figure 3. In previous studies (Lloyd-Fox et al., 2009) using similar experimental stimuli, the NIRS array was positioned over the temporal cortex. Here, however, the array was designed to allow measurement from a broad span of brain regions which included occipital, central, temporal and parietal regions in order to investigate coupling in different cortical areas that were expected to be activated by dynamic stimuli. Of the nine channels positioned over the right hemisphere, channels 14 and 15 (indicated in Figure 2) were located over the superior temporal sulcus – temporo-parietal (STS – TPJ) region, which previous studies have reported to exhibit the largest increase in brain activation to social stimuli (Lloyd-Fox et al., 2009).

### Data Analysis

All data was analysed in Matlab (Mathworks, United States) using in-house developed scripts. Wavelet-based motion correction (Molavi and Dumont, 2012) was first applied to the attenuation change signal of each participant, across all wavelengths, with tuning parameter α = 0.8, in order to correct for motion artefacts. The algorithm calculates wavelet coefficients for the signal using the discrete wavelet transform, which are assumed to have a Gaussian distribution. The coefficients that are determined to be outliers of the Gaussian distribution are then identified as artefacts and corrected. Following this, the UCLn algorithm (Bale et al., 2016) was used with a wavelength-dependent age-appropriate fixed differential path-length factor (DPF) value of 5.13 (Duncan et al., 1995) and 120 wavelengths between 780 and 900 nm were used in order to calculate changes in concentration of HbO_2_, HHb and ΔoxCCO. A 4th-order bandpass Butterworth filter from 0.01 to 0.4 Hz was used to filter the data, following which the data were segmented to create blocks consisting of 4 s of the baseline condition prior to the start of the experimental condition, the experimental condition, and the entire following baseline period. Baseline correction was performed. Following this, the video recordings of the infants from the testing session were used to code for looking-time offline. This involved identifying and removing trials during which the infant did not look at the experimental stimuli for an adequate duration. For this study, a trial was rejected if the infant did not look at the stimulus for at least 60% of the duration of the experimental stimulus. A minimum of two valid trials per condition were required for the infant to be included in the study. On average, one trial was removed due to looking-time for each infant. Only one infant was excluded for having insufficient number of trials based on looking-time.

Following the identification and rejection of trials of data based on looking time and the rejection of channels based on poor signal quality, for each infant, for each experimental condition, the changes in concentration of each of the chromophores were averaged across valid trials. This provided an average time-course response for each infant, for each chromophore. The responses for each infant were then averaged across participants in order to obtain a grand average response.

#### Further Exclusion Criteria

The photon counts (or intensity counts) from each of the detectors were used to assess signal to noise ratio and channels with counts lower than 2,000 or above 40,000 were excluded (Phan et al., 2016b). An infant was excluded from the study if they had more than 60% of channels excluded. Furthermore, at the group level, five channels over the occipital cortex were excluded due to poor signal quality in a majority of infants; Channel 3 (excluded in 64% of infants), Channel 6 (excluded in 83% of infants), Channel 7 (excluded in 64% of infants), Channel 8 (excluded in 79% of infants) and Channel 10 (excluded in 71% of infants). One channel over the temporal cortex (Channel 19) was excluded in 100% of participants due to a damaged optical fibre.

#### Statistical Analysis

A time-window of 14–18 s post-stimulus onset was selected for statistical analysis. A data driven approach was used for the selection of this time-window. This involved identifying the time at which the maximum or the “peak” of the response occurred across infants and channels and selecting a wide enough time-window to include the range of maximum concentration changes observed across infants for each of the chromophores. The response within the chosen time-window of 14–18 s was averaged, for each of the chromophores and this was used for statistical analysis.

After checking that the normality assumption was met, channel-wise one sample *t*-tests against 0 were conducted in order to test whether there was a statistically significant difference in the response to each of the experimental conditions from baseline. Following this, pairwise t-tests were then conducted to establish whether there was a statistically significant difference between the social and non-social conditions. The false discovery rate (FDR) procedure using the Benjamin and Hochberg method (Benjamini and Hochberg, 1995) was also performed in order to correct for multiple comparisons. Due to the exploratory nature of these analyses, results were reported for both un-corrected and corrected statistically significant responses.

#### Image Reconstruction

Image reconstruction was performed on the final, analysed dataset, at the individual subject level; images included in the Results section show data only from one randomly selected infant in order to provide an illustration of the capability of the image reconstruction analysis. For this analysis, three additional long-distance channels were created with source-detector separations of approximately 4.3 cm, these are shown in Figure 2. The reconstruction was performed only for channels over the right hemisphere as a number of channels over the occipital cortex were excluded and therefore an accurate image reconstruction could not be performed for these channels.

**FIGURE 4:**
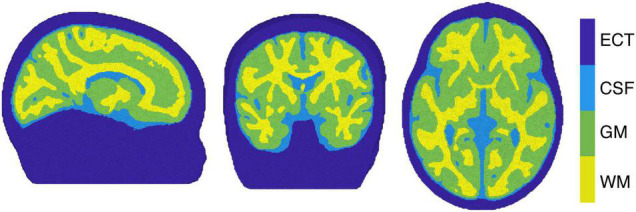
The tetrahedral mesh used for image reconstruction with white matter (WM), gray matter (GM), cerebrospinal fluid (CSF) and extra cerebral tissue (ECT) indicated.

The image reconstruction was performed by selecting the individual block averaged attenuation changes at 13 discrete wavelengths (from 780 to 900 nm at intervals of 10 nm) from the measured broadband data, to reduce the computational burden of the reconstruction while covering the available near-infrared spectrum. In addition, more than 8 wavelengths are required in order to reduce the error in estimating ΔoxCCO (Arifler et al., 2015). In this work, a four-layer infant head model – representing GM, WM, CSF and extra-cerebral tissue (ECT) – was built using averaged MRI data from a cohort of 12-month-old infants presented in Collins-Jones et al. (2021) and Shi et al. (2011). A four-layer voxelised model was initially constructed. Cerebral tissues in the model consisted of binary segmentations for GM, WM and CSF. The Betsurf segmentation procedure (Jenkinson et al., 2005) was then used to define an outer scalp boundary from the average head MRI template. All voxels that lay between the outer scalp boundary and the outer boundary of the CSF were defined as extra-cerebral tissue, a combined label for skull and scalp tissue. The voxelised four-layer model was converted to a high-resolution tetrahedral mesh, displayed in Figure 4 (∼7.8 × 10^5^ nodes and ∼4.7 × 10^6^ elements) using the iso2mesh software (Fang and Boas, 2009). The same software was used to create the GM surface mesh (∼5.8 × 10^4^ nodes and ∼1.2 × 10^5^ faces), used to display the reconstructed images.

Images of HbO_2_, HHb and ΔoxCCO were reconstructed as described elsewhere (Brigadoi et al., 2017), with a multispectral approach (Corlu et al., 2005), which directly reconstructs concentration changes from attenuation data. Wavelength-specific Jacobians were computed with the Toast++ software (Schweiger and Arridge, 2014) on the tetrahedral head mesh and projected onto a 50 × 60 × 50 voxel regular grid for reconstruction, using an intermediate finer grid of 100 × 120 × 100 voxels to optimise the mapping between mesh and voxel space. Optical properties were assigned to each tissue type and for each wavelength by fitting all published values for these tissue types (Bevilacqua et al., 1999; Strangman et al., 2002; Custo et al., 2006). Diffuse boundary sources and detectors were simulated as a Gaussian profile with a 2-mm standard deviation, and Neumann boundary conditions were applied. The inverse problem was solved employing the LSQR method to solve the matrix equations resulting from the minimisation and using first-order Tikhonov regularisation, with the parameter covariance matrix containing the diagonal square matrices with the background concentration values of the three chromophores (23.7 for HbO_2_, 16 for HHb and 6 for ΔoxCCO) (Zhao et al., 2005; Franceschini et al., 2007) and the noise covariance matrix set as the identity matrix. The maximum number of iterations allowed to the LSQR method was set to 50, and with a tolerance of 10^–5^. The regularisation hyperparameter λ was set to 10^–2^.

The reconstructed images, defined on the same regular grid of the Jacobian, were remapped to the tetrahedral head mesh and then projected to the GM surface mesh, by assigning a value to each node on the GM boundary surface that was equal to the mean value of all the tetrahedral mesh node values within a 3-mm radius. The concentration changes for HbO_2_ and HHb were normalised to the maximum concentration change of HbO_2_ while ΔoxCCO was normalised to its own maximum change in concentration.

#### Integration of Haemodynamic and Metabolic Data

For a detailed description of the method see Shokri-Kojori et al. (2019). Briefly, the rPWR and rCST are computed by performing a 45d° rotation of the haemodynamics and metabolism axes (see Fig1d-f in Shokri-Kojori et al., 2019). This creates a rPWR axis and a rCST axis. The rPWR axis signifies a match between metabolic demand and the observed haemodynamic activity (i.e., represents either a proportional concurrent increase or decrease in metabolic and haemodynamic activity), relative to the rest of the brain. The rCST axis represents the extent to which metabolic demand exceeds (or lags behind) the observed haemodynamic activity (i.e., represents a “mismatch” between metabolic and haemodynamic activity), relative to the rest of the brain.

Here, the mean response computed for statistical analysis in Statistical Analysis section was used (i.e., the mean response in the time-window of 14–18 s post-stimulus onset) and the rPWR and rCST values were computed for each channel and participant using the z-scored mean response values, across channels, for HbO_2_ and oxCCO (rPWR_HbO2_, rCST_HbO2_) and for HHb and oxCCO (rPWR_HHb_, rCST_HHb_). The sign for the HHb mean response was inverted so that the sign of rPWR_HHb_ and rCST_HHb_ matched that of rPWR_HbO2_ and rCST_HbO2_. In order to visualise the rPWR and rCST values at each channel, the z-scored mean response of ΔHbO_2_ or ΔHHb versus ΔoxCCO was plotted to obtain an understanding of which channels exhibited a match or mismatch between metabolism and haemodynamics.

In order to investigate patterns of rPWR and rCST in the infant brain in response to social and non-social stimuli, a one sample *t*-test against 0 was used to localise the brain regions which showed a significant match or mismatch between metabolic and haemodynamic activity, relative to the rest of the brain. Channel-wise *t*-tests versus 0 were performed at the group level for rPWR and rCST for HbO_2_ and HHb separately. FDR correction was performed to correct for the number of channels.

## Results

Out of the forty-two infants that participated in the study, data from twenty-five were included in the final analysis. Sixteen infants were excluded due to having more than 60% of channels with poor signal to noise ratio and 1 infant was excluded due to an insufficient number of trials for any of the conditions. This attrition rate is within the standard range for infant NIRS studies (Lloyd-Fox et al., 2010).

### Social and Non-social Conditions

**FIGURE 5:**
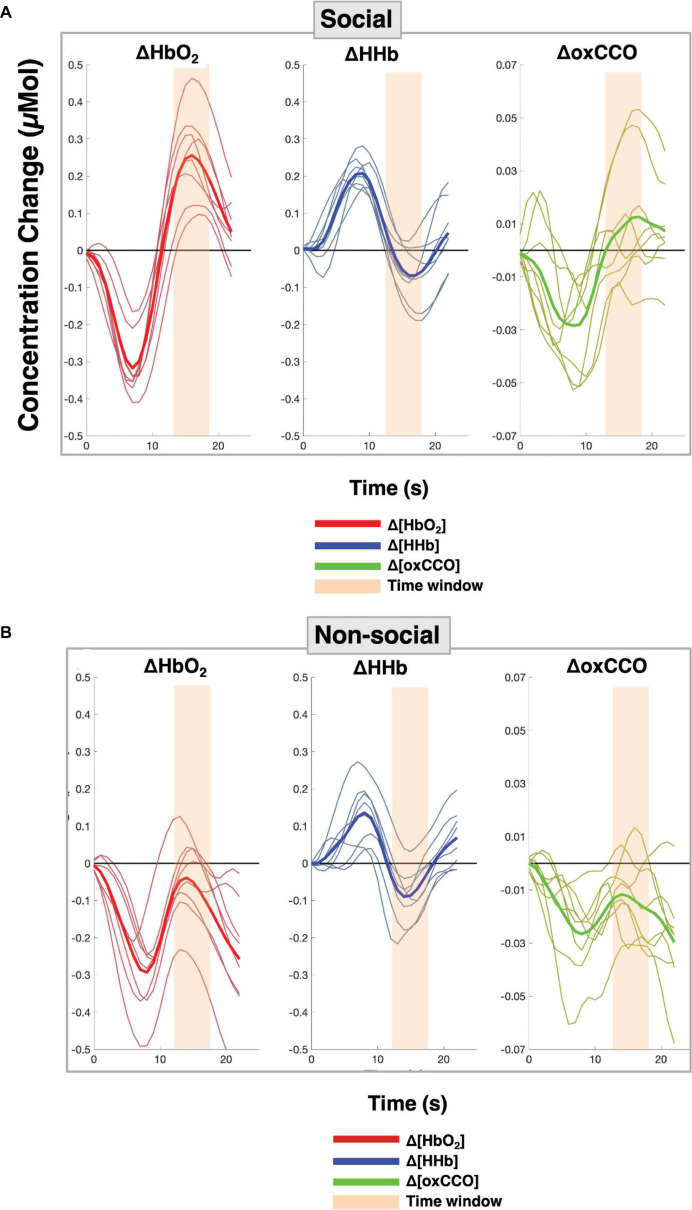
Grand averaged time courses for each of the chromophores with each red, blue and green line representing the response per channel and the solid line indicating the grand average across all channels over the right hemisphere for **(A)** the Social condition and **(B)** the Non-social condition.

Figure 5 shows the grand averaged time courses for the social and non-social conditions, for all the channels over the right hemisphere. The time courses from the individual channels are shown in the Supplementary Material.

### Image Reconstruction

The results from the image reconstruction can be found in the Supplementary Material.

### Integration of Haemodynamic and Metabolic Data

**FIGURE 6:**
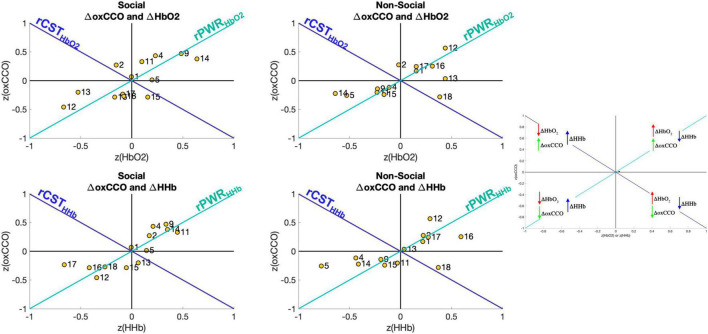
Visual representation of rPWR and rCST for the Social condition (Left) and the Non-Social condition (Right). These parameters were computed from the mean response in the time-window 14–18 s post-stimulus onset (the same time-window used for statistical analysis) which were z-scored across channels and consequently averaged across participants, for oxCCO and HbO_2_ (Top) and oxCCO and HHb (Bottom) by rotating 45° along the haemodynamics and metabolism axes, yielding an rPWR axis (teal) and the rCST axis (blue). The map is divided into four quadrants based on the direction and relative magnitude of the chromophores’ changes (greater increase or decrease across channels).

To evaluate the relationship between brain haemodynamics and metabolism, the rPWR and rCST parameters were calculated by combining the mean response in the 14–18 s post-stimulus onset time-window of ΔHbO_2_ or ΔHHb with the mean response of ΔoxCCO. Figure 6 shows the haemodynamics versus metabolism plot for both the social and non-social conditions. Here, the figure is divided into four quadrants based on the magnitude and direction of the changes in HbO_2_/HHb and oxCCO. For example, the channels in the top right quadrant exhibit a greater increase in HbO_2_ and oxCCO and a greater decrease in HHb which leads to a high rPWR value. Here, Channels 9 and 14 in particular, for the Social condition, have a high rPWR value while Channel 12 has a high rPWR value for the Non-Social condition. This implies that for the Social condition, brain areas over which Channels 9 and 14 are located have a concurrent change in haemodynamics and metabolism in response to the stimulus. Meanwhile for the Non-Social condition, Channel 12 displays concurrent haemodynamic and metabolic activity. The majority of channels are located in the top right and bottom left quadrants (implying that there is a positive or a negative rPWR value at that channel and hence a match between haemodynamic and metabolic activity). However, some channels lie in the top left and bottom right quadrants (implying that there is a positive or negative rCST value at that channel and hence a mismatch between haemodynamic and metabolic activity). This is particularly evident for Channels 2 and 15 for the Social Condition for HbO_2_ and Channel 13 for HHb and Channel 18 for the Non-Social Condition.

### Regional Cortical Specialisation to Social and Non-social Stimuli

**FIGURE 7:**
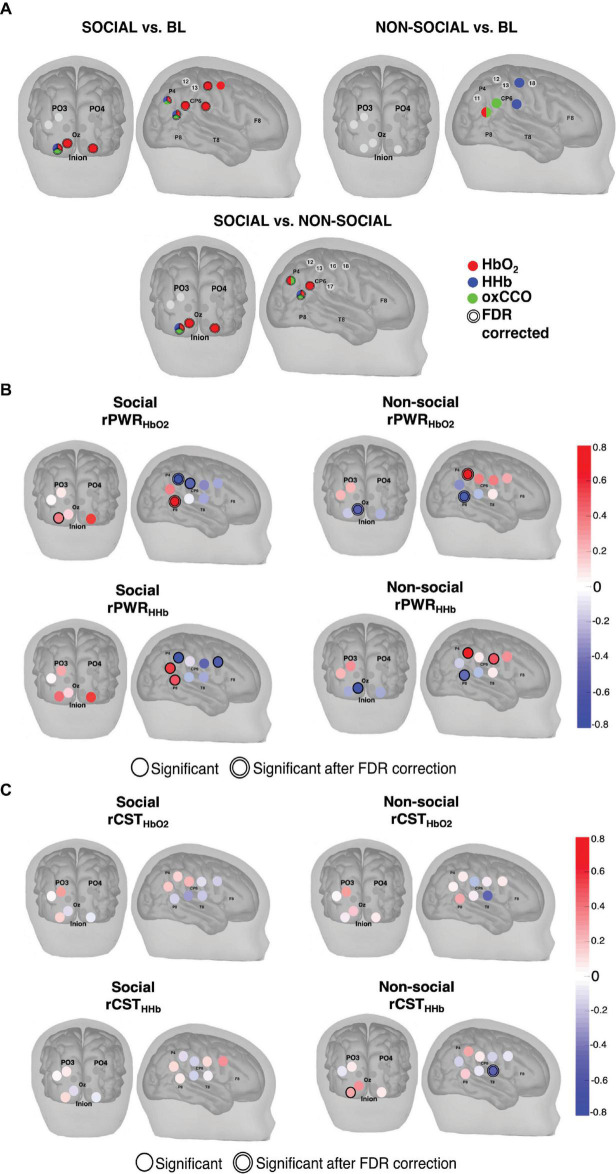
**(A)** Channels with statistically significant responses for (Top, Left) the Social condition versus baseline, (Top, Right) the Non-social condition versus baseline and (Bottom) the Social versus the Non-social condition for HbO2 (red), HHb (blue) and ΔoxCCO (green). The double line around the channel indicates statistical significance after FDR correction. **(B)** Activation maps resulting from the group-level one sample *t*-test against 0 performed on the Social condition rPWR values (Top, Left) for HbO_2_ and (Bottom, Left) for HHb and the Non-Social condition rPWR values (Top, Right) for HbO_2_ and (Bottom, Right) for HHb. **(C)** Activation maps resulting from the group-level one sample *t*-test against 0 performed on the Social condition rCST values (Top, Left) for HbO_2_ and (Bottom, Left) for HHb and the Non-Social condition rCST values (Top, Right) for HbO_2_ and (Bottom, Right) for HHb. Shaded circles represent the rPWR or rCST value and a single black line represents significance (*p* < 0.05) while a double black line indicates significance after FDR correction A positive rPWR value implies a positive association between oxCCO and HbO_2_/HHb. This means that relative to the other channels, channels with a positive rPWR have a greater concurrent increase in HbO_2_ and oxCCO and a decrease in HHb and an increase oxCCO. A negative rPWR value corresponds to a negative association between oxCCO and HbO_2_/HHb. This means that relative to other channels, channels with a negative rPWR have a greater concurrent decrease in HbO_2_ and oxCCO and a greater increase in HHb and decrease in oxCCO. Relative to the other channels, channels with a positive rCST are those where metabolic activity exceeds haemodynamic activity. Channels with a negative rCST are those where haemodynamic activity exceeded metabolic activity.

Combining the two approaches described above, Figure 7 shows the results from both the statistical analysis comparing social and non-social conditions and the rPWR/rCST analysis. Figures 7A–C indicates channels with a statistically significant response to the social and non-social conditions versus baseline as well as the social versus the non-social condition for HbO_2_ (red), HHb (blue) and ΔoxCCO (green). The results indicate a stronger response to the social condition for both HbO_2_ and ΔoxCCO in comparison to the non-social condition. The comparison between the social and non-social conditions (Figure 7C) shows a more localised response to the social condition for ΔoxCCO (significant activation in 25% of channels) in comparison to HbO_2_ (significant activation in 50% of channels). In order to identify patterns of localised coupling between haemodynamics and metabolism, channel-wise one sample *t*-tests were carried out against 0 on rPWR and rCST for HbO_2_ and HHb. Group-level results for rPWR and rCST are shown in Figures 7E–L with rPWR and rCST values mapped for both Social and Non-social conditions. A distinct spatial pattern of coupling between haemodynamics and metabolism can be seen with posterior parieto-temporal channels showing a concurrent and proportional increase in haemodynamic and metabolic activity for the Social condition and the central-parietal channels showing a concurrent increase for the Non-social condition.

## Discussion

We used multichannel broadband NIRS to obtain the first spatially resolved, non-invasive, simultaneous measures of cerebral cellular energy metabolism and oxygenation/haemodynamics during functional activation in infants. Our results demonstrated that all broadband NIRS measures (Δ[HbO_2_], Δ[HHb] and Δ[oxCCO]) display significant differences in the processing of social and non-social stimuli. Further investigation of the relationship between bNIRS measures with the rPWR and rCST measures revealed spatially specific, cortical specialisation to social and non-social stimuli.

Naturalistic stimuli designed to be ecologically valid for this age group (dynamic social versus non-social movies) were used. In response to the social video, we observed a widespread increase in Δ[HbO_2_] in channels over the right hemisphere which was accompanied by a more localised concurrent increase in Δ[oxCCO] and a decrease in Δ[HHb] in channels over the STS-TPJ region. Over the occipital cortex, for the social condition, an increase in Δ[HbO_2_] and ΔoxCCO was observed, along with a decrease in Δ[HHb], in the ventral/lateral occipital channels. In comparison to the baseline, there was a significant increase in Δ[HbO_2_] in response to the social condition in 69% of all channels (60% of channels over the occipital cortex and 75% of channels over the right hemisphere) while there was significant increase in ΔoxCCO in 23% of all channels (20% of channels over the occipital cortex and 25% of channels over the right hemisphere. This might indicate distinct spatial localisation of the cytochrome response in comparison to the haemodynamics. Studies have demonstrated that task-related increases in oxidative metabolism and energy demand were smaller (approximately 15%) in comparison to the increase in CBF (approximately 60%) (Lin et al., 2010; Bruckmaier et al., 2020), which could potentially explain the fewer number of channels activated for ΔoxCCO. Indeed, previous studies have suggested that oxygen consumption is more spatially specific in comparison to changes in cerebral blood flow (Malonek and Grinvald, 1996). Further, adult fNIRS studies measuring responses over the visual cortex have demonstrated distinct spatial distributions of ΔoxCCO and Δ[HbO_2_] (Phan et al., 2016a). The distribution of the cytochrome oxidase enzyme has previously been mapped histochemically in the human visual cortex (Wong-Riley et al., 1993) and the rat brain (Hevner et al., 1995). These studies provide evidence that energy metabolism may be spatially specific.

For the non-social condition, fewer channels over the right hemisphere showed a significant difference from the baseline for all three chromophores. Unexpectedly, no channels over the occipital cortex showed a significant difference from baseline, for any of the chromophores as basic visual processing of the stimuli was expected. These results replicate earlier findings that report that differential responses to social and non-social dynamic naturalistic stimuli can be seen in early infancy (Farroni et al., 2013). Moreover, the investigation of coupling between the bNIRS measures revealed distinct, spatially specific and stimulus-dependent patterns of matched haemodynamic and metabolic activity. For the social condition, a significant coordinated increase in haemodynamic and metabolic activity (i.e., positive rPWR) was observed over the STS-TPJ region and a significant coordinated decrease (i.e., negative rPWR) over the parietal region. Interestingly, these associations were reversed for the non-social condition, i.e., positive rPWR was observed over the parietal region and negative rPWR over the STS-TPJ region. Taken together, these results indicate that not only are the measured haemodynamic changes accompanied by measurable changes in cellular energy metabolism, but that the concurrent measurement of these signals provides us with a better understanding of stimulus-driven spatially localised activity and early cortical specialisation.

### Regionally Specific, Stimulus-Dependent Networks of Matched Haemodynamic and Metabolic Activity

While the statistical analysis indicated spatial patterns of activation for social and non-social conditions, limited information could be inferred about the relationship between metabolic and haemodynamic activity. Gaining an understanding of this relationship is important to understand cortical specialisation. Shokri-Kojori et al. (2019) proposed a method, which derived measures (rPWR and rCST), to investigate coordination/coupling between cerebral energy metabolism and haemodynamic activity and demonstrated better brain network differentiation with the rPWR/rCST measures. Briefly, rPWR represents coordinated metabolic and haemodynamic activity and rCST represents the extent to which metabolism exceeds haemodynamic activity. We employed this method and found an emerging pattern of specialised brain function in infancy.

Our results revealed regional networks where a coordinated increase in haemodynamic and metabolic activity could be observed. Different brain regions exhibited coordinated activity for the social and non-social stimuli. For the social stimuli, haemodynamic and metabolic activity was most strongly coordinated in channels over the ventro-lateral occipital cortex, in accordance with previous fMRI work (Deen et al., 2017), and in channels over the posterior STS-TPJ (a coordinated increase in haemodynamics and metabolism was observed in these channels). Given that the social stimuli were a combination of faces and biological motion, these results are consistent with previous studies which have demonstrated that the aforementioned brain regions are involved in networks associated with face-processing (referred to as the ventral face network) (Grill-Spector et al., 2017) as well as processing of biological motion (Grossman and Blake, 2002). Meanwhile for the non-social condition, in contrast to the social condition, there was a coordinated decrease in haemodynamic and metabolic activity over the ventral occipital channels, but a pattern of positive association (i.e. coordinated increase) was observed over dorsal occipital channels. In addition, channels over the parietal and central regions showed a stronger coordinated increase in metabolic and haemodynamic activity while those over the temporal regions showed a coordinated decrease. These results are in line with previous research which has demonstrated that the parietal cortex plays an important role in object processing (Wilcox et al., 2010; Dekker et al., 2011; Jeong and Xu, 2016). Most interestingly, the aforementioned patterns of matched haemodynamic and metabolic activity could not be observed through the bNIRS time-course responses or the statistical analysis. These results emphasise the importance of combined haemodynamic and metabolic measurements to identify task-specific areas of brain activation. More specifically, these combined measurements have the potential to unveil brain regions that are specialised for the processing of different stimuli with a higher spatial specificity.

### Brain Regions With a Mismatch in Haemodynamic and Metabolic Activity

The majority of channels over the occipital cortex and the right hemisphere showed coordinated haemodynamic and metabolic activity. However, the results showed that there were certain brain regions where haemodynamics and metabolism were mismatched. Specifically, a significant mismatch was observed over the parietal-central area for the non-social condition, where haemodynamic activity appeared to exceed metabolic activity as reflected by negative rCST (which represents the extent to which metabolic activity exceeds haemodynamic activity). This is in contrast to the results presented in a previous paper that utilised the same technique with broadband NIRS measurements over the visual cortex in adults (Pinti et al., 2021), where all cortical regions showed coordinated/matched cerebral metabolism and haemodynamic activity. In contrast, our results show that specific channels have a mismatch (see Figure 6). This potentially indicates that while overall, haemodynamics and metabolism are coupled in infancy, this relationship (particularly in specific cortical regions) is still developing. Previous studies in rats have demonstrated that during brain development, coupling between cerebral haemodynamics and metabolic changes are not fully established and that functional hyperaemia matures gradually as cerebral vasculature develops alongside cortical connectivity (Arichi et al., 2012; Kozberg et al., 2013; Kozberg and Hillman, 2016). This is particularly important to consider given that many infant NIRS studies report heterogeneity in the infant response to functional activation, with some studies reporting an increase in Δ[HbO_2_] and a decrease in Δ[HHb] (Taga et al., 2004; Wilcox et al., 2005; Bortfeld et al., 2007; Lloyd-Fox et al., 2014) and some studies reporting the opposite, i.e., a decrease in Δ[HbO_2_] and an increase in Δ[HHb] in response to an experimental stimulus (Csibra et al., 2004; Telkemeyer et al., 2009; Kobayashi et al., 2011; Watanabe et al., 2012). There may be multi-factorial reasons leading to the observed heterogeneity in responses, which may well include regional cortical developmental vascular and structural changes as well as maturation of neurovascular coupling (Issard and Gervain, 2018). A combined analysis of metabolism and haemodynamics can greatly aid us in gaining a better understanding of the development of these mechanisms in infancy.

### Conclusion

This work demonstrates the use of multichannel broadband NIRS in 4-to-7-month-old infants to obtain spatially resolved measures of ΔoxCCO alongside haemodynamic changes. This study provides the first measurements that allow the exploration of the relationship between cerebral energy metabolism and haemodynamic changes in the developing human brain. The results suggest that ΔoxCCO may be a more spatially specific marker of neuronal activation and that concurrent measurement of haemodynamics and metabolism has the potential to yield vital information about cortical specialisation in infancy. To our knowledge, this is the first reported use of multichannel broadband NIRS to map the spatial distribution of the ΔoxCCO response and its relationship to haemodynamics in early infancy. Measuring cellular energy metabolism in infants will greatly aid our understanding of basic biological mechanisms in the developing human brain and in particular, their contribution in brain specialisation. One area in which this may be particularly useful is in understanding atypical brain development. Neurodevelopmental disorders are known to affect metabolism, and specifically, autism spectrum disorders (ASD) have been linked to mitochondrial dysfunction (Watanabe et al., 2008). Using broadband NIRS, the measurement of changes in metabolic activity, alongside haemodynamics could further our understanding of how alterations in energy metabolism and neurovascular coupling mechanisms may result in apparent differences in neural processing, which are observed in neurodevelopmental disorders such as autism (Zhu et al., 2014; Lloyd-Fox et al., 2017; Braukmann et al., 2018). Moreover, this technique can be highly valuable in investigated age-related brain maturation processes and their association with developmental milestones.

### Limitations

Due to technical limitations, channels were placed over the right hemisphere and the occipital cortex which did not allow for comparison of responses over the right and left hemispheres. Moreover, the temporal resolution of the bNIRS system was 1 s, which is slower in comparison to traditional fNIRS systems. Future bNIRS systems will have higher acquisition rate for faster sampling.

## Data Availability Statement

The data is available upon reasonable request and subject to a formal data sharing agreement.

## Ethics Statement

The studies involving human participants were reviewed and approved by Birkbeck Ethics Committee. Written informed consent to participate in this study was provided by the participants’ legal guardian/next of kin.

## Author Contributions

MS conducted the study. MS, SL-F, EJ, CE, and MJ developed the protocols for the study. IT provided the NIRS system and support with data acquisition. MS, PP, SB, and LC-J analysed the data with support from SL-F, EJ, CE, and MJ. MS wrote the manuscript with support from EJ, IT, CE, SL-F, and MJ. All authors contributed to the article and approved the submitted version.

## Conflict of Interest

The authors declare that the research was conducted in the absence of any commercial or financial relationships that could be construed as a potential conflict of interest.

## Publisher’s Note

All claims expressed in this article are solely those of the authors and do not necessarily represent those of their affiliated organizations, or those of the publisher, the editors and the reviewers. Any product that may be evaluated in this article, or claim that may be made by its manufacturer, is not guaranteed or endorsed by the publisher.
