# Supplementary material for: Regional Haemodynamic and Metabolic Coupling in Infants
Source: Front Hum Neurosci. 2022 Feb 4;15:780076. doi: 10.3389/fnhum.2021.780076 (PMC8854371; doi:10.3389/fnhum.2021.780076)
Supplement: Supplementary file 1 [file Data_Sheet_1.DOCX]

Supplementary Material

# Image reconstruction

## Methods

Image reconstruction was performed on the final, analysed dataset, at the individual subject level; images included in the results section show data only from one randomly selected infant in order to provide an illustration of the capability of the image reconstruction analysis. For this analysis, three additional long-distance channels were created with source-detector separations of approximately 4.3cm. The reconstruction was performed only for channels over the right hemisphere as a number of channels over the occipital cortex were excluded and therefore an accurate image reconstruction could not be performed for these channels.

The image reconstruction was performed by selecting the individual block averaged attenuation changes at 13 discrete wavelengths (from 780 to 900 nm at intervals of 10 nm) from the measured broadband data, to reduce the computational burden of the reconstruction while covering the available near-infrared spectrum. In addition, more than 8 wavelengths are required in order to reduce the error in estimating ΔoxCCO (Arifler, Zhu, Madaan, & Tachtsidis, 2015). In this work, a four-layer infant head model – representing GM, WM, CSF and extra-cerebral tissue – was built using averaged MRI data from a cohort of 12-month-old infants presented in Shi et al. (2011). A four-layer voxelised model was initially constructed. Cerebral tissues in the model consisted of binary segmentations for GM, WM and CSF. The Betsurf segmentation procedure (Jenkinson, Pechaud, & Smith, 2005) was then used to define an outer scalp boundary from the average head MRI template. All voxels that lay between the outer scalp boundary and the outer boundary of the CSF were defined as extra-cerebral tissue, a combined label for skull and scalp tissue. The voxelised four-layer model was converted to a high-resolution tetrahedral mesh (∼7.8 × 10^5^ nodes and ∼4.7 × 10^6^elements) using the iso2mesh software (Fang & Boas, 2009). The same software was used to create the GM surface mesh (∼5.8 × 10^4^ nodes and ∼1.2 × 10^5^ faces), used to display the reconstructed images.

Images of HbO_2_, HHb and ΔoxCCO were reconstructed as described elsewhere (Brigadoi et al., 2017), with a multispectral approach (Corlu et al., 2005), which directly reconstructs concentration changes from attenuation data. Wavelength-specific Jacobians were computed with the Toast++ software (Schweiger & Arridge, 2014) on the tetrahedral head mesh and projected onto a 50 × 60 × 50 voxel regular grid for reconstruction, using an intermediate finer grid of 100 × 120 × 100 voxels to optimize the mapping between mesh and voxel space. Optical properties were assigned to each tissue type and for each wavelength by fitting all published values for these tissue types (Bevilacqua et al., 1999; Custo, Wells, Barnett, Hillman, & Boas, 2006; Strangman, Culver, Thompson, & Boas, 2002). Diffuse boundary sources and detectors were simulated as a Gaussian profile with a 2-mm standard deviation, and Neumann boundary conditions were applied. The inverse problem was solved employing the LSQR method to solve the matrix equations resulting from the minimization and using first-order Tikhonov regularization, with the parameter covariance matrix containing the diagonal square matrices with the background concentration values of the three chromophores (23.7 for HbO_2_, 16 for HHb and 6 for ΔoxCCO) (Franceschini et al., 2007; Zhao, Ding, Hou, Zhou, & Chance, 2005) and the noise covariance matrix set as the identity matrix. The maximum number of iterations allowed to the LSQR method was set to 50, and with a tolerance of 10^-5^. The regularization hyperparameter λ was set to 10^-2^.

The reconstructed images, defined on the same regular grid of the Jacobian, were remapped to the tetrahedral head mesh and then projected to the GM surface mesh, by assigning a value to each node on the GM boundary surface that was equal to the mean value of all the tetrahedral mesh node values within a 3-mm radius. The concentration changes for HbO_2_ and HHb were normalised to the maximum concentration change of HbO_2_ while ΔoxCCO was normalised to its own maximum change in concentration.

## Results

Supplementary Figure 1 shows the image reconstructions from an individual infant at three time-points: stimulus onset, 10 s post-stimulus onset and 18 s post-stimulus onset, for both social and non-social conditions. In accordance with the time-courses shown in the Figures 2 and 3 (in the supplementary information), for the social condition, an initial decrease in Δ[HbO_2_] and Δ[oxCCO] and an initial increase in Δ[HHb] is observed. This is followed by a subsequent increase in Δ[HbO_2_] and Δ[oxCCO] and a decrease in Δ[HHb]. Meanwhile for the non-social condition, a decrease in Δ[HbO_2_] and Δ[oxCCO] can be observed. The videos of the image reconstructions have also been included as supplementary material.


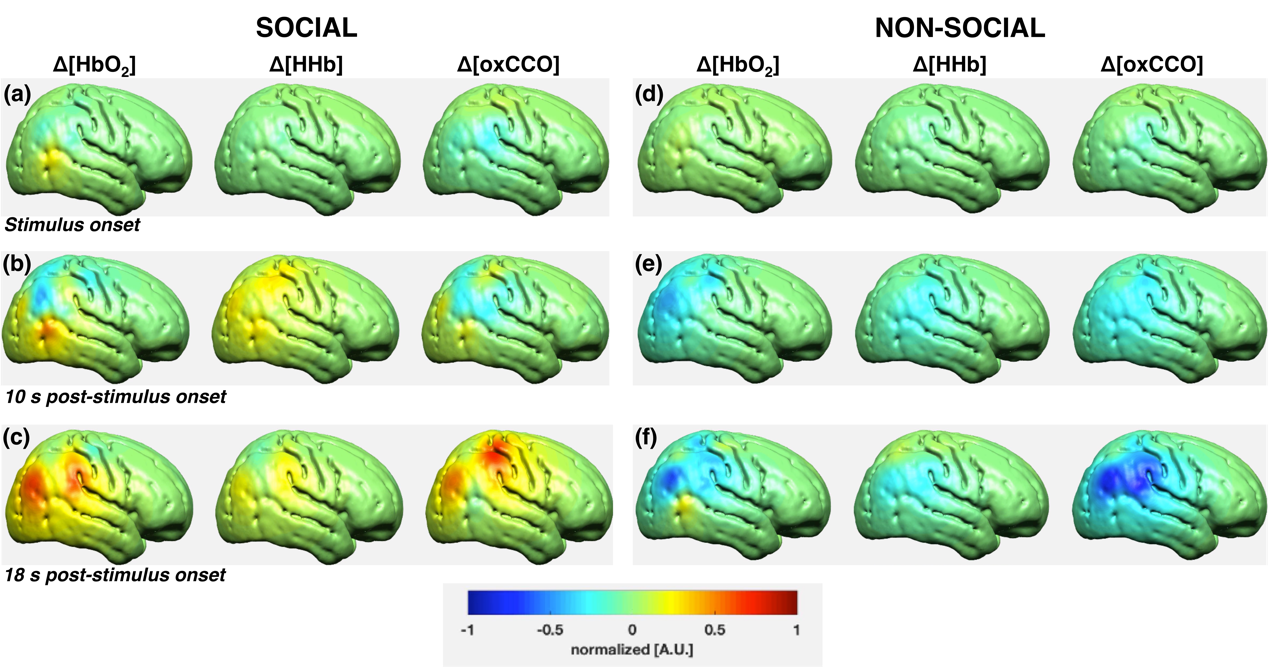


Supplementary Figure 1: Image reconstructions from a single infant at three specified time points; stimulus onset, 10 s post-stimulus onset and 18 s post-stimulus onset for (a) – (c) the social condition and (d) – (f) the non-social condition with HbO_2_ shown on the left, HHb in the centre and ΔoxCCO on the right. The concentration changes for HbO_2_ and HHb were normalised to the maximum concentration change of HbO_2_ while ΔoxCCO was normalised to its own maximum change in concentration.

# Social and Non-social conditions

## Results

Figures 2 and 3 show the grand averaged time courses (n=25) from each of the channels over the right hemisphere for both the social and non-social conditions respectively. Larger changes in Δ[HbO_2_] and ΔoxCCO can be seen for the social condition in comparison to the non-social condition, with ΔoxCCO showing a fewer number of channels with significant activation in comparison to Δ[HbO_2_]. Table 1 shows the *p*-values and t-values from each channel for both conditions.





Supplementary Figure 2: Grand averaged changes in concentration of HbO_2_ (red), HHb (blue) and ΔoxCCO (green) at each of the channels over the occipital cortex and the right hemisphere for the Social condition. The scale on the left is for Δ[HbO_2_] and Δ[HHb] while the scale on the right is for ΔoxCCO. The coloured box indicates the time window used for statistical analysis. The coloured circles indicate a statistically significant response to the condition (prior to FDR correction) versus the baseline for HbO_2_ (red), HHb (blue) and ΔoxCCO (green) and a double line indicates significance after FDR correction.





Supplementary Figure 3: Grand averaged changes in concentration of HbO_2_ (red), HHb (blue) and ΔoxCCO (green) at each of the channels over the occipital cortex and the right hemisphere for the Non-Social condition. The scale on the left is for Δ[HbO_2_] and Δ[HHb] while the scale on the right is for ΔoxCCO. The coloured box indicates the time window used for statistical analysis. The coloured circles indicate a statistically significant response to the condition (prior to FDR correction) versus the baseline for HbO_2_ (red), HHb (blue) and ΔoxCCO (green) and a double line indicates significance after FDR correction.
